# Behavioural and electrophysiological (event-related potential) determinants of decision-making in individuals with a smoking habit

**DOI:** 10.3389/fpsyg.2026.1743972

**Published:** 2026-03-25

**Authors:** Julien Dampuré, Paola Agudelo-Orjuela, Maartje Van der Meij, Horacio A. Barber, David Belin

**Affiliations:** 1 Laboratoire sur les Processus Psychologiques et Sociaux (2PS), Université Catholique de l'Ouest, Niort, France; 2 Instituto Universitario de Neurociencia (IUNE), Universidad de La Laguna (ULL), Tenerife, Spain; 3 Departamento de Psicología Cognitiva, Universidad de La Laguna (ULL), Tenerife, Spain; 4 Department of Physiology, Development and Neuroscience, University of Cambridge, Cambridge, United Kingdom

**Keywords:** addiction, decision making, event-related potentials (ERP), Iowa gambling task (IGT), P300—event related potential

## Abstract

**Introduction:**

Individuals with a smoking habit or with other addictions display deficits in decision-making, which are suggested to contribute to the perpetuation of their compulsive behaviour. In individuals with a smoking habit (ISH), performance in the Iowa Gambling Task (IGT), which operationalises real-life decision making under uncertainty, is worsened by both exposure to craving-inducing cigarette-related cues and abstinence. However, the behavioural and neurophysiological nature of “baseline” decision-making in ISH, the impairment of which may reflect a general insight deficit, related to an increased reliance on implicit motivational mechanisms, such as incentive habits, in controlling behaviour, has not been fully elucidated.

**Methods:**

Here, we sought to characterise the behavioural and electrophysiological (Event-Related Potentials, ERPs) determinants of “baseline” decision-making, as assessed in the IGT, in 20 ISH compared to 20 control participants. Neurophysiological processes related to option evaluation, response selection and feedback integration were assessed as the pre-outcome N500 (480–520 ms), the DNP and the post-outcome FRN & fP300 (450–700 ms) ERPs across five blocks of 60 trials during which the selection of advantageous decks, an index of decision making in the IGT, was recorded.

**Results:**

While ISH did not perform significantly less well than controls overall under these baseline conditions, they displayed only half the improvement in decision making shown by controls over the session, and they engaged different neurophysiological processes. While the N500 amplitude was more negative for non-risky than for risky choices across groups, it was slightly smaller in ISH than in controls. Similarly, while the amplitude of the FRP was greater for negative than for positive outcomes across groups, in ISH, this contrast was inversely proportional to the severity of the smoking habit, as assessed with the Fagerström test.

**Discussion:**

These findings together identify biobehavioural determinants of decision making in individuals with a smoking habit that may represent an endophenotype of the impaired insight that characterises individuals with a substance use disorder.

## Introduction

Patients with a substance use disorder (SUD) display a lack of behavioural awareness linked to drug use-related behaviours, alexithymia, diminished sensitivity to somatic markers of risk, and reduced capacity to predict the long-term consequences of choices (Maracic and Moeller, 2021; Paulus and Stewart, 2014). These behavioural manifestations reflect impairments in insula-dependent interoceptive insight (Naqvi and Bechara, 2009; Belin et al., 2011; Goldstein et al., 2009), otherwise necessary for the accurate integration and monitoring of bodily signals to guide adaptive behaviour (Bechara and Damasio, 2005).

In individuals with a SUD, these insight deficits have been suggested together with impairments in top-down executive control (Zilverstand et al., 2018) to promote the expression of maladaptive incentive drug-seeking habits and the associated aberrant influence drug-paired cues have on behaviour that contribute to poor decision making, the perpetuation of addictive behaviours in the face of adverse consequences (Maracic and Moeller, 2021; Goldstein et al., 2009; Zilverstand et al., 2018; Noel et al., 2013; Bechara, 2005; Olsen et al., 2015; Bechara et al., 2006; Belin et al., 2013; Ramey and Regier, 2019; Watson et al., 2012) and relapse, even after long periods of abstinence (Clark and Robbins, 2002; Claus et al., 2011; American Psychiatric Association, 2013).

For instance, in individuals with an alcohol use disorder or a smoking habit (ISH), performance in prefrontal cortex (PFC)-dependent inhibitory control or decision-making tasks (Konishi et al., 1999; Chikazoe, 2010; Sasaki et al., 2023) is worsened by both exposure to craving-inducing cues and abstinence (Campanella et al., 2020; Detandt et al., 2017; Mitchell, 2004; Miglin et al., 2017). At the neural systems level, cigarette-related cues have been shown to modulate both response-selection-related P3 and response-evaluation-related Pc components in an emotional Go-No Go task in ISH but not in control, non-regular smoker (NRS) participants (Dampure et al., 2023). Critically, in ISH, the P3-Go facilitation effect towards smoking-related cues involved the activation of the posterior cingulate cortex (PCC), which has also been shown to be conjointly engaged with the insula for representations of emotional/somatic states that bias choices in decision-making tasks under uncertainty (Li et al., 2010). This is in line with long-established evidence that drug-related cues exert a bidirectional effect on the quick, intuitive, emotional and automatic affective-automatic and the slow, rational and conscious reflective-deliberative systems, eventually biasing control over decision making and behaviour by the former (McClure and Bickel, 2014). This suggests that in SUD, dysfunction within the PFC–PCC–insula circuitry contributes to sub-optimal utilisation of somatic markers to guide decision-making, thereby amplifying the reliance on habitual, cue-driven responding. Accordingly, in ISH, smoking-related cues selectively weaken inhibitory processing and promote habitual responding, reflecting the long-term influence of conditioned reinforcement on automatic behaviour (Dampure et al., 2023). Thus, in individuals with SUDs drug-paired cue presentations or abstinence, which trigger cognitively taxing urges of negative affective valence (Hillebrand, 2000), impair decision making and promote habitual responding (Dampure et al., 2023; Garbusow et al., 2014). However, since both cue-induced craving and abstinence engage specific interoceptive, emotional and cognitive mechanisms that may overshadow a constitutive alteration in the neurophysiological processes otherwise involved in decision making under neutral conditions, henceforth referred to as “baseline” (Moran-Santa Maria et al., 2015; Mattioni et al., 2024; Bell and Froeliger, 2021), the behavioural and neurophysiological nature of such “baseline” decision-making in ISH has not been fully elucidated. This is particularly surprising because impairments in baseline decision making in ISHs are likely to reflect a general, constitutive, insight deficit with an increased reliance on maladaptive implicit motivational mechanisms (Tiffany, 1990), such as rigid incentive habits (Belin et al., 2013).

In the Iowa Gambling Task (IGT), which operationalises real-life decision making under uncertainty, individuals with SUD often show poor performance compared to control participants (Ariesen et al., 2023; Gowin et al., 2018; Businelle et al., 2008). In this task, participants have to choose a card from one of four decks on each trial, with the goal of maximising their monetary gains. Two of the decks (C and D) are advantageous, as they yield low immediate gains, but overall, fewer losses than gains over time. The other two decks (A and B) are initially more appealing, but clearly disadvantageous as they lead to larger immediate rewards but even larger cumulative losses overall. While at the population level, individuals tend initially to choose the appealing options, they progressively move away from these to select more and more frequently the advantageous decks over trials. Individuals with SUD usually present a lower tendency to choose advantageous cards than control participants, displaying a lower percentage of advantageous choices overall, or a lower net score, calculated as the number of cards drawn from the advantageous decks (Decks C and D) minus those selected from the disadvantageous decks (Decks A and B; Barry and Petry, 2008; Verdejo-Garcia et al., 2007). This behavioural impairment has been shown at the neurophysiological level to be accompanied by attenuated somatosensory- and feedback-related potentials (Fein and Chang, 2008; Stewart et al., 2013), consistent with a compromised neural integration of bodily insight to guide higher-order decision processes.

Neurophysiological investigations of the processes involved in decision making, such as those engaged in the IGT, have usually reported that two main ERP components are sensitive to the valence and/or magnitude of the outcome (Latibeaudiere et al., 2025; Euser et al., 2011), namely the FRN and the feedback P300 (fP300). The FRN is, an early fronto-central negative deflection generated by the ACC that peaks 200–300 ms after feedback (Amiez et al., 2005; Bellebaum and Daum, 2008). The FRN has been shown to be larger for negative or unexpected outcomes than for positive or expected outcomes, thereby reflecting the rapid evaluation of the affective and motivational significance of the outcome necessary to guide learning. The fP300 is a positive component that follows the FRN, peaking about 300–600 ms after feedback. With losses generally eliciting larger fP300 amplitudes than gains, due to their salience or unexpectedness, this ERP has been suggested to reflect attentional allocation, working memory updating, and high-level evaluation of feedback. Together, the FRN and fP300 likely capture distinct stages of outcome processing, with the former representing rapid affective appraisal and prediction errors, and the latter reflecting a subsequent evaluation of the functional significance of the feedback. Individuals with with Internet Addiction (Balconi et al., 2017), Internet Gaming Disorder (Raiha et al., 2020), heroin (Zhao et al., 2017) or methamphetamine use disorder (Wei et al., 2021) are all characterised by altered FRN and fP300, indicating an impaired sensitivity to negative feedback. For instance, in the Balloon Analogue Risk Task (BART; Lejuez et al., 2002), in which participants inflate a balloon to accumulate gains, with each additional pump increasing the risk of the balloon bursting and losing the accumulated reward, adolescent smokers displayed altered feedback-related potentials, including FRN latency, that were associated with the severity of nicotine use (Morie et al., 2021). Conversely, individuals with a SUD tend to generate fP300 of larger amplitude for gains than losses (Euser et al., 2011; Balconi et al., 2017; Zhou et al., 2010; Wei et al., 2018), alongside an enhanced activity of the ventral striatum during reward outcome as compared to individuals without SUD (Luijten et al., 2017). Altogether, these findings suggest that FRN and fP300 modulations may reflect differential processing of feedback valence, which can be altered in populations with reward-related dysfunctions.

In marked contrast, the SUD-specific neurophysiological signature of the processes occurring prior to the execution of the response in a decision-making situation, e.g., during the card selection phase in the IGT, has not been investigated. This stage likely reflects the interplay of two closely intertwined mechanisms, namely the option evaluation and response selection (Latibeaudiere et al., 2025). Electrophysiological studies have highlighted choice-dependent modulations of specific ERP components, particularly the P300 and N500, during the option evaluation stage of the IGT. The P300 component evoked between 300 and 600 ms in the centroparietal areas shows a larger positive amplitude when participants choose a card from a disadvantageous or risky deck compared to one from an advantageous or safe deck, reflecting its association with working memory processes and the motivational or emotional significance of the stimuli (Guo et al., 2019). Then, a negative ERP component peaking around 500 ms after stimulus onset, known as the N500, exhibits a greater magnitude for risky choices. This has been associated with higher-order cognitive control and conflict monitoring processes (Botelho et al., 2023; Yang et al., 2007; Yang et al., 2010), reflecting the neural mechanisms engaged in evaluating advantageous vs. disadvantageous options. Finally, some studies have reported a component known as the Decision Preceding Negativity (DPN), a slow cortical wave emerging approximately 800 ms before response selection, with larger negative amplitudes preceding disadvantageous deck choices (Bianchin and Angrilli, 2011). Source localisation studies have identified the right superior frontal gyrus as a key generator of this component (Giustiniani et al., 2015), a region implicated in evaluating the consequences of choices and in intuitive judgement processes. The DPN is thought to reflect anticipatory mechanisms engaged before risky decisions, preparing the neural system to mobilise avoidance or approach behaviors (Latibeaudiere et al., 2025; Bianchin and Angrilli, 2011; Dong et al., 2016; Cui et al., 2013). Its right-lateralized activity aligns with the involvement of the frontal cortex in processing negative emotions and regulating affective responses to potential risks (Morawetz et al., 2019). Importantly, while the DPN indexes general risk anticipation, previous research suggests it does not directly account for individual differences in strategic performance, highlighting its role as a preparatory rather than performance-determining signal (Giustiniani et al., 2015).

To the best of our knowledge, the neurophysiological (ERPs) correlates of choice evaluation, response selection and feedback processing in nicotine-only smokers have not been characterised (but see 50 for multi-drug, including nicotine, users). Thus, in the present study smokers (ISH) and never-smokers (NRS) performed a classical IGT, while ERPs associated with the choice evaluation stage (P300, N500), response selection (DPN) and feedback evaluation stage (FRN, fP300) were recorded. We predicted, according to the somatic marker hypothesis framework of economic decision (Bechara and Damasio, 2005), that smokers, with a relatively lower interoceptive insight (Belin et al., 2011; Mattioni et al., 2024) than NRS, would perform less well than the latter in the IGT. In particular, we expected the gap between net scores, overall lower in ISH than NRS, to widen over the course of the session as the latter, progressively adopt an optimal strategy. At the neurophysiological level, we expected a DPN of greater amplitude for disadvantageous than for advantageous choices during the response selection process, as previously shown. During the choice evaluation stage, we anticipated to observe disadvantageous choices to evoke more positive P300 and/or more negative N500 than advantageous choices. Finally, we anticipated these ERPs to be modulated by the smoking status, namely being greater in ISH than NRS.

Since individuals with SUDs display both a heightened sensitivity to rewards and a diminished sensitivity to negative outcomes (Potts et al., 2014), we predicted that ISH would exhibit a reduced amplitude difference between positive and negative outcome conditions for either the FRN or the fP300, which are highly sensitive to feedback valence, thereby reflecting a potential electrophysiological biomarker of impaired interoceptive insight. Finally, while nicotine use severity has been reported to modulate feedback-related ERPs in cannabis users performing a decision-making task (i.e., the Balloon Analogue Risk Task; Morie et al., 2021), whether this effect is driven by nicotine, cannabis, or their interaction remains unclear. Thus, a last objective of the present study was to determine whether ERPs associated with the feedback evaluation stage are shaped by the severity of nicotine dependence, as assessed by the Fagerström Test (Heatherton et al., 1991). We hypothesised that the magnitude of the reduction of the amplitude difference between positive and negative outcome conditions for either the FRN or the fP300 expected in ISH would be proportional to the severity of their addiction.

## Materials and methods

### Participants

The same sample of participants as described in (Dampure et al., 2023) was used for the present study. It consisted of 40 individuals residing in Tenerife (Spain), including 20 regular smokers (15 women; mean age = 30.8 ± 12.4 years) and 20 individuals who had never smoked (15 women; mean age = 30.0 ± 12.3 years). All participants were Spanish nationals, recruited through convenience sampling in the towns of La Laguna and Santa Cruz de Tenerife. They provided informed consent prior to participation and received financial compensation (€15) for completing the study. The research protocol was approved by the Ethical Committee of the University of La Laguna (CHUC_2018_04 PAB16NT, Tenerife, Spain).

Demographic information (e.g., gender, age), clinical background, and neuropsychological profiles of participants were collected via an online questionnaire administered through the PsyToolkit platform (Stoet, 2010). Smokers were additionally evaluated on their smoking behaviour using the Fagerström Test for Nicotine Dependence and the Obsessive-Compulsive Smoking Scale (OCSS), allowing for the classification of their smoking status as light-to-moderate (Schane et al., 2010; Wilson et al., 1999). All participants were screened for psychiatric or substance use disorders via a brief clinical interview, and the presence of any ongoing medication was recorded. Carbon monoxide (CO) levels were measured prior to the experimental task using a Smokerlyzer (Bedford Scientific Ltd., Rochester, United Kingdom), and smokers reported their spontaneous craving levels both before and after the session on a 31-point scale.

As detailed in (Dampure et al., 2023), smokers and never-smokers did not significantly differ in their mood or anxiety levels. However, smokers had higher impulsivity scores [69 ± 10.8 vs. 58 ± 8.8, *F*_1,38_ = 11.19, *p* < 0.002, *_p_η^2^* = 0.23] and significantly elevated CO levels [14.05 ± 9.32 ppm vs. 4.75 ± 1.45 ppm, *F*_1,38_ = 18.76, *p* < 0.0001, *_p_η^2^* = 0.33] compared to never-smokers.

Although relatively small, this sample was shown to be sufficient to reveal subtle neurophysiological differences with large effect sizes between controls and ISH in a modified Go/NoGo task (Dampure et al., 2023).

### Apparatus

The experimental stimuli were displayed on a 17-inch monitor with a resolution of 768 × 1,024 pixels, using the E-Prime 2.0 software (Psychology Software Tools), and were controlled by a personal computer. Electroencephalographic (EEG) data were recorded via the EasyCap system (BrainVision), using 27 Ag/AgCl electrodes placed according to the international 10–20 system, with reference to the left mastoid. To monitor eye movements, four supplementary electrodes were used: two positioned at the outer corners of the eyes to capture horizontal electro-oculographic (EOG) activity, and two others placed above and below the right eye to monitor vertical EOG. The EEG signal was amplified and recorded within a frequency range of 0.01 to 100 Hz, at a sampling rate of 500 Hz. Electrode impedance was maintained below 5 kΩ, and below 10 kΩ for the EOG channels.

### Materials

#### Task paradigm and procedure

A modified version of the Iowa Gambling Task (IGT) was employed to assess decision-making under conditions of uncertainty. The task, which was the same as the original version except that it was administered in Spanish and used euros as currency (as opposed to dollars in the original version), with slightly higher monetary gains and losses to adjust for real-life value while preserving the original structure of the task. It involved four decks of cards (A, B, C, and D), two of which were advantageous (C and D), while the remaining two (A and B) were disadvantageous. Decks A and B were classified as disadvantageous due to their negative average outcomes (−76€ and −170€, respectively). Deck A involved frequent but relatively moderate losses (up to −465€), whereas deck B featured infrequent but substantial losses (up to −2,150€). In contrast, decks C and D yielded positive long-term returns (average gains of 42€ and 52€, respectively), with lower-magnitude losses (up to −145€ and −1,000€, respectively).

**Figure 1:**
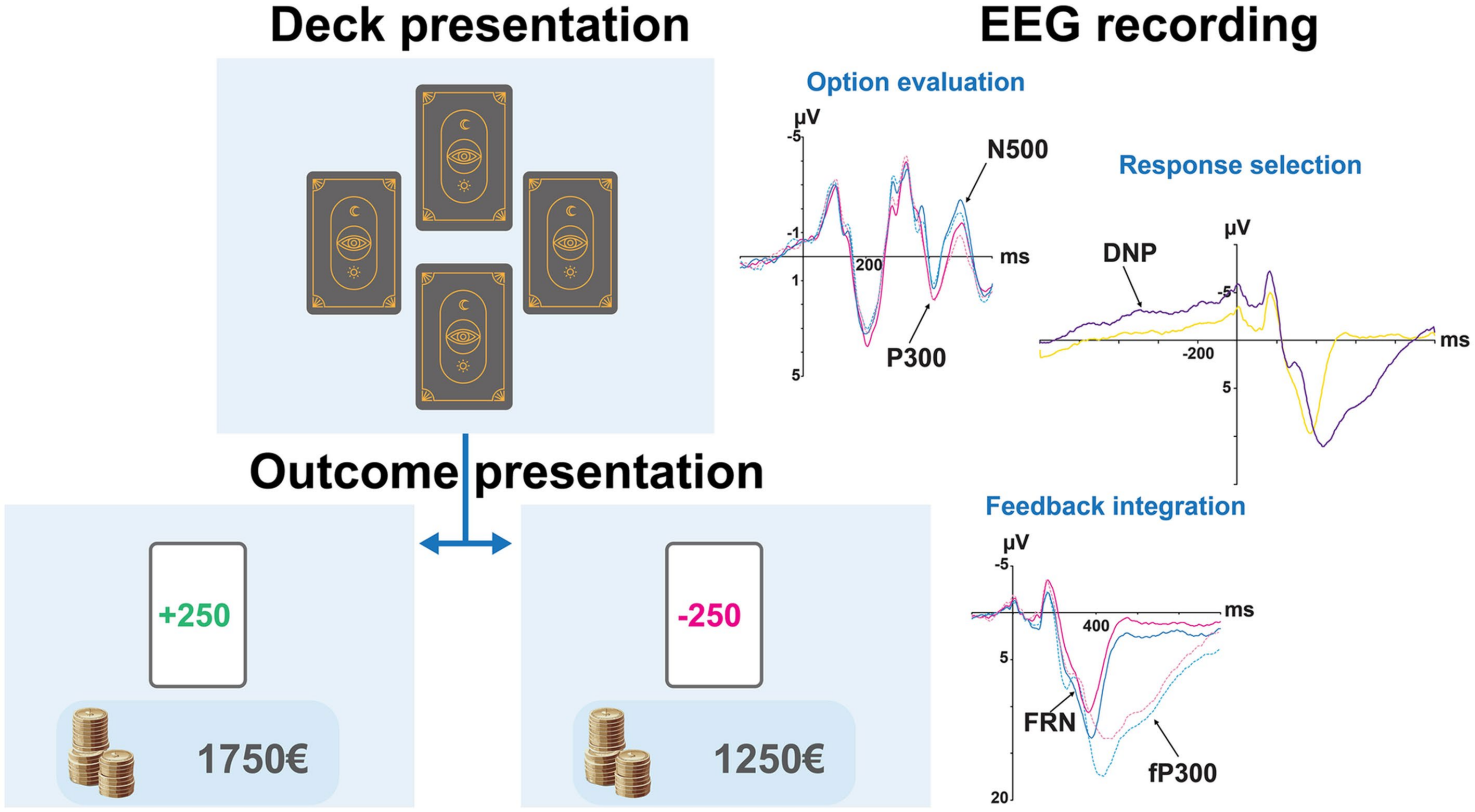
Experimental procedure of the Iowa Gambling Task (IGT) and associated event-related potentials (ERPs). The left side of the figure illustrates the different stages of the Iowa Gambling Task (IGT) used in the experiment, starting with the deck presentation followed by the outcome presentation, during which feedback indicated either a monetary gain or a loss, with cumulative earnings displayed below the outcome card. The EEG recording (right side) illustrates the main ERP components associated with each stage: option evaluation (P300, N500), response selection (decision-related negativity, DNP), and feedback integration (feedback-related negativity, FRN, and feedback P300, fP300).

Participants completed a total of 300 trials. Participants began the task with a balance of 0€ and were instructed to maximise their long-term gains. Each trial began with the presentation of the four desks of cards. Card selection was made using a Microsoft Xbox controller, with the X, Y, A, and B buttons corresponding spatially to the on-screen positions of decks A, B, C, and D, respectively (see Figure 1). Immediately after button press, they received feedback for 1,000 ms indicating the amount gained or lost, along with the cumulative total earned since the beginning of the task.

#### EEG preprocessing and data analysis

EEG data were processed offline using Brain Analyser software (Brain Products GmbH, Gilching, Germany). A band-pass filter ranging from 0.1 to 30 Hz was applied to the recorded signal. The data were then re-referenced to the average activity of the two mastoid electrodes, and ocular artefacts were corrected using Independent Component Analysis (ICA; Makeig and Jung, 1995; Jung et al., 1998). Trials containing artefacts were manually excluded, with comparable rejection rates observed between smokers and controls (3.6 and 2.7%, respectively, *t*_38_ = 0.76, *p* = 0.45).

Subsequently, a dedicated processing pipeline was implemented to extract event-related potentials (ERPs) time-locked similarly for all the participants to three distinct task events:

1. Choice evaluation stage: This stage corresponded to the onset of deck presentation and was used to analyse two ERP components: the pre-outcome P300 (390–450 ms), associated with motivational and emotional processing, and the N500 (480–520 ms), linked to conflict monitoring and risky decision-making. For each segmentation, a 200 ms pre-stimulus baseline was applied, and average ERP waveforms were computed separately for disadvantageous/risky (DisO) and advantageous/safe choices (AdvO);
2. Response selection stage: The analysis focused on the Decision-Related Negativity (DNP; −800 to 0 ms), which has been associated with the anticipation of risky decisions. EEG epochs were centred on the response onset, with a − 1,000 ms to +1,000 ms window used for baseline correction (Carlson, Zayas and Guthormsen, 2009) and average ERP waveforms were computed separately for risky and safe choices;
3. The feedback stage: The analysis focused on the Feedback Related Negativity (FRN, 300–360 ms), which reflects rapid evaluation of the affective and motivational significance of the outcome, which was identified as the most negative peak following the subtraction of the ERPs evoked by negative *minus* positive outcomes; and the feedback P300 (fP300; 450–700 ms), which reflects the motivational evaluation of outcomes. For each segmentation, a 200 ms pre-stimulus baseline was applied, and average ERP waveforms were computed separately for positive and negative outcomes.

Following the procedure described in Dampure et al. (2023), the EEG data were processed through the following steps for each of the three periods:

1. Averages per participant were computed for each segmentation within the standardised dedicated time windows, determined based on the literature.
2. Grand averages were then calculated, and the maximal peak amplitude was determined within each standardised dedicated time window;
3. The mean peak amplitudes within each standardised dedicated time window were exported and analysed.

Decision-making efficiency in the IGT was assessed by calculating the number of advantageous choices within five blocks of 60 trials across the total 300-trial session. Repeated-measures Analyses of Variance (ANOVAs) were conducted using JASP software, following verification of the assumptions of normality (via Shapiro–Wilk test) and homogeneity of variance (via Mauchly’s test). However, since sphericity was violated, the Greenhouse–Geisser correction was applied to these ANOVAs. The number of advantageous choices was compared using Group (ISH vs. NRS) as a between-subject factor, and Trial Block (0–60, 61–120, 121–180, 181–240, 241–300) as a within-subjects factor. To investigate the potential contribution of impulsivity to IGT performance, an analysis of covariance (ANCOVA) was conducted using the same design as the original ANOVA, with BIS scores as a covariate.

In order better to understand the differential processes driving decision-making in ISH and NRS, an ANOVA was conducted on the amplitude of the P300 (390 to 450 ms) from one side of the brain and the N500 (480 to 520 ms) from the contralateral side, taking the type of choice (advantageous vs. disadvantageous), the Region (frontal, frontocentral, centroparietal), Laterality (left, central, right), and Electrode (e1, e2, e3) as within-subject factors, and the Group of participants (ISH vs. NRS) as between-subject factor. Accordingly, the following electrodes were included: frontal left (F7, FC5, F3), frontal midline (Fp1, Fz, Fp2), frontal right (F8, FC6, F4), frontocentral right (T8, CP6, C4), frontocentral midline (FC1, Cz, FC2), frontocentral left (T7, CP5, C3), centroparietal right (P7, P3, O1), centroparietal midline (CP1, Pz, CP2), centroparietal left (P8, P4, O2).

Upon confirmation of significant main effects, differences among individual means were analysed using the Scheffé post-hoc test. For all analyses, significance was set at *α* = 0.05 and effect sizes were reported as partial eta squared ($\eta_{p}^{2}$) for every significant effect.

As an exploratory approach, a cortical source estimation was conducted using LORETA to further characterise the effects observed within each ERP component of interest (P300, N500, DNP, FRN and fP300), i.e., delineate the cortical territories showing maximal activation within each component’s standardised time window.

## Results

### Characteristics of the experimental groups

Individuals with a smoking habit had a smoking history and scores in the Fagerstrom test and the OCSS characteristic of light-to-moderate smokers (Schane et al., 2010; Wilson et al., 1999; see Table 1). They showed an increase in their spontaneous level of craving following the experimental session [16.2 ± 10.9 vs. 18.42 ± 10.3, *F*_1,19_ = 6.23, *p* = 0.02, $\eta_{p}^{2}$ = 0.25] before which they had a much higher level of carbon monoxide than NRS controls [14.05 ± 9.32 ppm vs. 4.75 ± 1.45 ppm, *F*_1,38_ = 18.76, *p* < 0.0001, $\eta_{p}^{2}$ = 0.33], from whom they did not differ in their anxiety, and mood levels, as assessed by the STAI, Beck and PANAS. However, ISH were more impulsive than NRS controls, as revealed by a much higher score in the BIS (*t_38_* = 3.35, *p* = 0.002, *d* = 1.06; Table 1).

**Table 1:** Demographic and personal data of individuals with a smoking habit and non-regular smoker controls.

Variable	Individuals with a smoking habit	NRS Controls	*p*-value
Age	30.8 ± 12.4	30 ± 12.3	ns
Smoking history	12.5 ± 10.7	N/A	N/A
Fagerstrom test	4.1 ± 2.4	N/A	N/A
OCSS	23.8 ± 5.8	N/A	N/A
BIS-11	69 ± 10.8	58 ± 8.8	0.002
STAI-R	26.5 ± 9.3	21 ± 11.5	ns
Beck	21.1 ± 14.2	2.75 ± 11.8	ns
PANAS+	28.35 ± 5.8	29.75 ± 7.5	ns
PANAS-	20.35 ± 8.7	18.25 ± 8.2	ns

### Behavioural data

**Figure 2:**
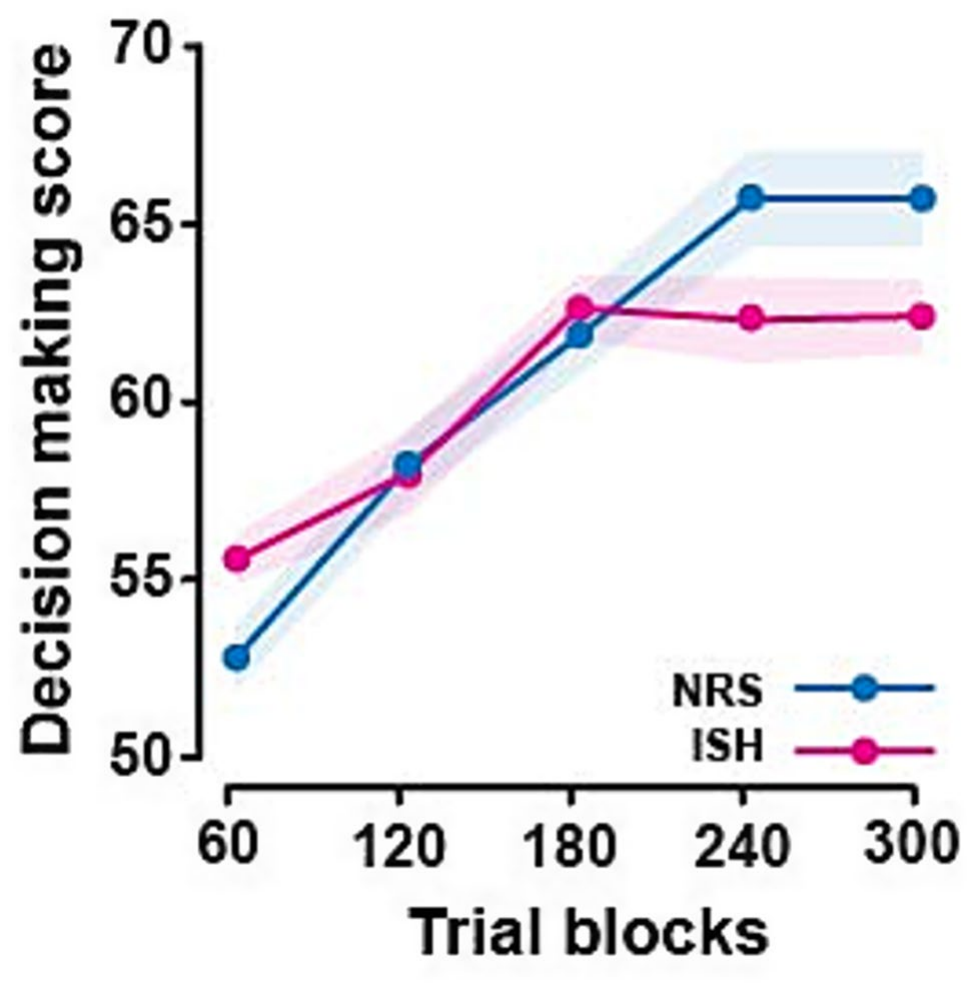
Individuals with a smoking habit performed less well than non-regular smoker controls in the Iowa Gambling Task. NRS (non-regular smoker) control participants showed a statistically significant 23% increase in their selection of advantageous decks over the five 60-trial blocks of the task. This was twice as much as individuals with a smoking habit (ISH), who displayed a non-statistically significant increase of only 11.69%.

During the IGT, overall, the participants increasingly selected the more advantageous desks over the five blocks of 60 trials (main effect of block: *F*_3,117_ = 3.37, *p* = 0.01, $\eta_{p}^{2}$ = 0.08; see Table 2). However, such a progressive shift away from the more appealing, but disadvantageous options towards the less appealing but advantageous ones occurred in NRS but not in smokers, as revealed by simple main effects (*F*_3,117_ = 2.65, *p* = 0.04, $\eta_{p}^{2}$ = 0.07 and *F*_3,117_ = 0.99, *p* = 0.42, respectively; Figure 2).

**Table 2:** *Post-hoc* comparisons (with Holm correction) between blocks of 60 trials.

Block i	Block j	Mean Difference	SE	t	Cohen’s d	p_holm_
1	2	−3.550	2.995	−1.185	−0.182	1
	3	−7.300	2.995	−2.437	−0.375	0.128
	4	−8.900	2.995	−2.971	−0.457	0.033
	5	−8.950	2.995	−2.988	−0.459	0.033
2	3	−3.750	2.995	−1.252	−0.192	1
	4	−5.350	2.995	−1.786	−0.275	0.514
	5	−5.400	2.995	−1.803	−0.277	0.514
3	4	−1.600	2.995	−0.534	−0.082	1
	5	−1.650	2.995	−0.551	−0.085	1
4	5	−0.050	2.995	−0.017	−0.003	1

IGT performance overall or between groups cannot be accounted for by impulsivity trait or the higher levels of impulsivity shown by ISH as demonstrated by an ANCOVA using BIS score as co-variate (Main effect of impulsivity on IGT performance: *F*_1,37_ = 0.114, *p* = 0.74; and learning across blocks: Block × BIS interaction: *F*_3.06,113.07_ = 0.51, *p* = 0.683; Group × BIS interaction: *F*_1,38_ = 0.001, *p* = 0.99; Group × Block × BIS interaction: *F*_3.07,116.69_ = 0.47, *p* = 0.711, Greenhouse–Geisser corrected). Overall, BIS scores explained less than 1% of the variance ($\eta_{p}^{2}$ = 0.003), confirming the negligible contribution of impulsivity to IGT performance.

### ERP results

#### Option evaluation stage

**Figure 3:**
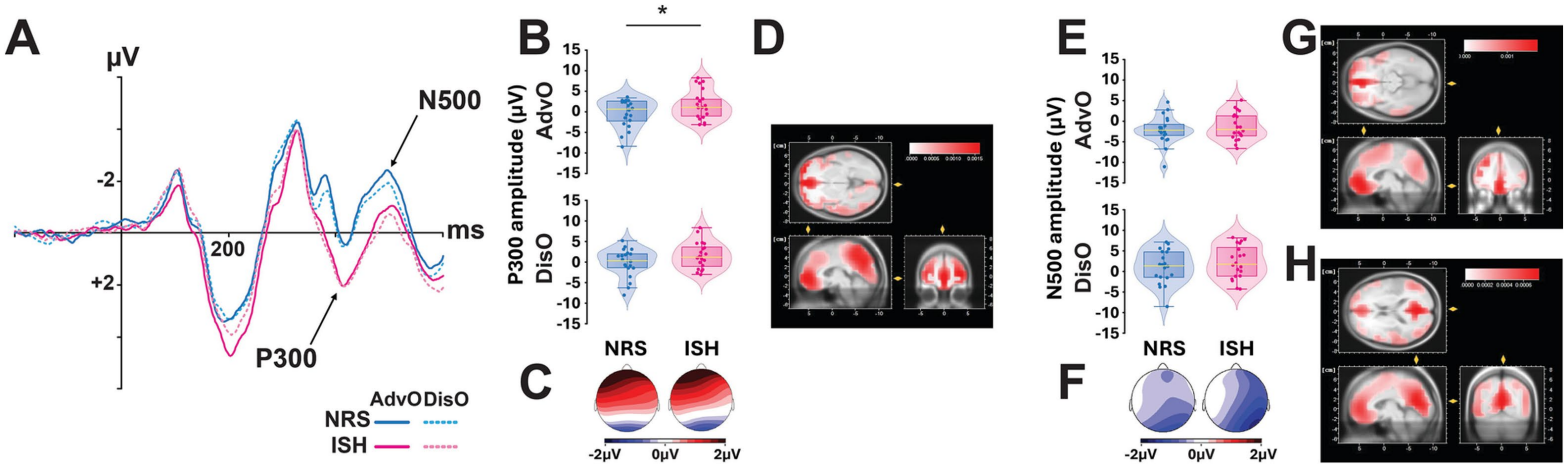
Individuals with a smoking habit exhibited ERPs that differed from those elicited by non-regular smoker controls during the option evaluation stage. **(A)** Event-related potentials (ERPs) recorded at Cz for advantageous (AdvO, solid line) and disadvantageous (DisO, dotted line) options are shown for non-regular smoker controls (NRS, blue) and individuals with a smoking habit (ISH, pink). **(B)** Boxplots depict the mean P300 voltage (μV) at Cz within the 390–450 ms time window, accompanied by the corresponding scalp topographies **(C)**. *p < 0.05. ISH elicited larger P300 amplitudes than NRS **(A,B)**. This effect was associated with activity in the orbitofrontal cortex (OFC), as revealed by LORETA **(D)**. **(E)** Boxplots depict the mean N500 voltage (μV) at Cz within the 480–520 ms time window, accompanied by the corresponding scalp topographies **(F)**. LORETA-based cortical source estimations for the outcome effect suggest distinct cortical activation patterns in ISH (orbitofrontal cortex, OFC) compared to NRS (posterior cingulate cortex, PCC) (G, H).

P300 (390–450 ms) amplitudes (Figures 3A–D) were more positive in the centroparietal region than in either the frontocentral (*t*_76_ = 4.56, *p* < 0.001, *d* = 0.52) or frontal (*t*_76_ = 6.73, *p* < 0.001, *d* = 0.77) regions even though those on the former were greater than in the latter (*t*_76_ = 2.17, *p* < 0.05, *d* = 0.25). These regional differences in P300 amplitudes were predicated on the smoking status of the participants (*F*_2,76_ = 5.17, *p* = 0.008, $\eta_{p}^{2}$ = 0.12; Figures 3A,B) in that ISH exhibited larger P300 amplitudes than NRS in the frontal (*F*_1,76_ = 4.36, *p* = 0.044, $\eta_{p}^{2}$ = 0.05), but not the frontocentral (*F*_1,76_ = 1.50, *p* = 0.23) or centroparietal (*F*_1, 76_ = 0.36, *p* = 0.55) regions.

The smoking status did not shape the influence of the type of choice or laterality of the amplitude of the P300 (*F*_2,76_ = 11.26, *p* < 0.001, $\eta_{p}^{2}$ = 0.23; Figure 3C). Simple main effects revealed that disadvantageous/risky choices elicited more positive P300 than advantageous choices in the right hemisphere (*F*_1,76_ = 6.19, *p* = 0.017, $\eta_{p}^{2}$ = 0.08) but not in the left (*F*_1,76_ = 3.52, *p* = 0.33) or the midline (*F*_1,76_ = 0.03, *p* = 0.86) region. This lateralised response was not uniform across regions (*F*_4,152_ = 3.25, *p* = 0.014, $\eta_{p}^{2}$ = 0.08) with P300 magnitudes differing between choices in the centroparietal (*F*_1,152_ = 8.44, *p* = 0.006, $\eta_{p}^{2}$ = 0.05) and frontocentral (*F*_1,152_ = 4.84, *p* = 0.034, $\eta_{p}^{2}$ = 0.03) regions but not in frontal region (*F*_1,152_ = 2.35, *p* = 0.13). Exploratory source estimation using LORETA suggested that the influence of smoking status on the P300 amplitudes was related to a differential activation of the Orbitofrontal cortex (OFC, see Figure 3D), the intensity of which was estimated to be greater in ISH than in NRS.

The type of choice also influenced the amplitude of the N500 (480 to 520 ms; Figures 3E,F; *F*_1,38_ = 7.03, *p* = 0.01, $\eta_{p}^{2}$ = 0.16), which was more negative for advantageous than for disadvantageous choices (−0.67 μV ± 1.91 and −0.15 μV ± 2.19), respectively (Figures 3A–E). Like for the P300, this effect varied across regions (*F*_2,76_ = 6.93, *p* = 0.002, $\eta_{p}^{2}$ = 0.15, Figure 3F). Planned comparisons demonstrated that the N500 magnitude was greater for advantageous than for disadvantageous choices in the Centroparietal (*t*_53_ = 3.88, *p* < 0.001, *d* = 1.07) and Frontocentral (*t*_53_ = 2.06, *p* = 0.045, *d* = 0.57) regions, but not in the Frontal region (*t*_53_ = 1.33, *p* = 0.19). These differences were also lateralised (*F*_2,76_ = 11.63, *p* < 0.001, $\eta_{p}^{2}$ = 0.23). The greater N500 amplitude following advantageous choices was observed predominantly on the right side (*t*_49_ = 3.96, *p* < 0.001, *d* = 1.13) and the central part of the scalp (*t*_49_ = 2.49, *p* = 0.01, *d* = 0.71), but not on the left side (*t*_49_ = 0.98, *p* = 0.33). This effect was predicated on the smoking status (*F*_2,76_ = 2.99, *p* = 0.05, $\eta_{p}^{2}$ = 0.07), in that ISH had a marginally smaller -though not statistically significant- advantageous choice-related N500 than NRS on the left side; as revealed by simple main effects (*F*_1,76_ = 3.73, *p* = 0.061, $\eta_{p}^{2}$ = 0.05; all other *Ps* > 0.16).

An exploratory LORETA cortical source estimation during the N500 time window further suggested that the neural substrates of the two types of choice (advantageous vs. disadvantageous) differed by smoking status. As shown in Figures 3G,H, this activity was estimated to originate in the orbitofrontal cortex (OFC) in ISH but in the posterior cingulate cortex (PCC) in NRS.

#### Response selection stage

**Figure 4:**
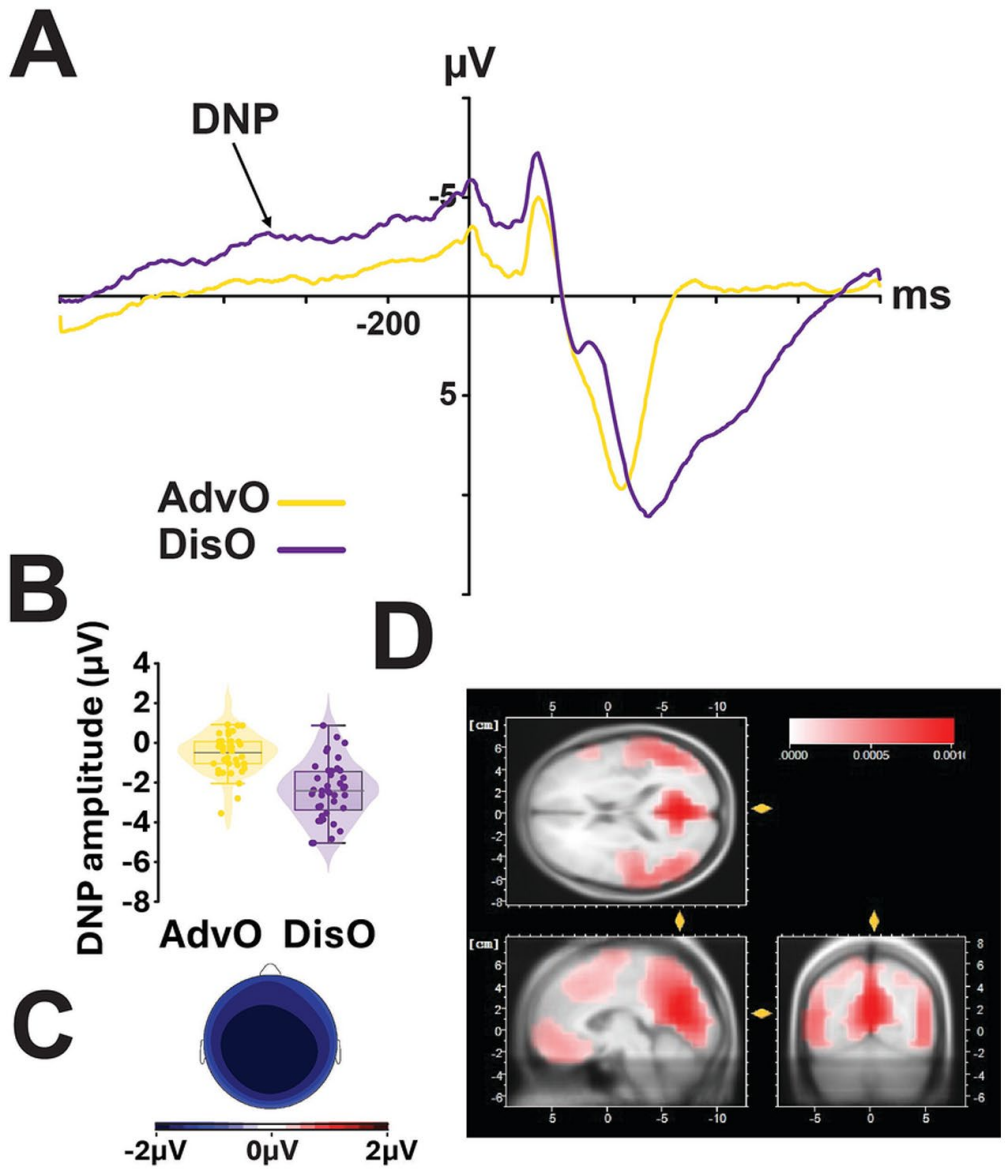
Decision preceding negativity (DPN, −800–0 ms) during the response selection stage was larger before disadvantageous (risky) choices, irrespective of the smoking status of the individual. **(A)** Event-related potentials (ERPs) recorded at Fz for disadvantageous (DisO, purple) and advantageous (AdvO, yellow) outcomes. **(B)** Boxplots depict the mean DPN voltage (μV) at Fz within the −800 to 0 ms time window, accompanied by the corresponding scalp topographies **(C)**. Disadvantageous or risky choices elicited more negative DPNs than advantageous or safe choices, independently of smoking status. Additionally, cortical source estimations using LORETA **(D)** suggest a preferential involvement of the posterior cingulate cortex (PCC).

The DPN amplitude (Figures 4A,B) was influenced by the type of choice (*F*_1,38_ = 81.37, *p* < 0.001, $\eta_{p}^{2}$ = 0.68), being greater before a risky than a safe choice, but not by smoking status (*F*_1,38_ = 1.97, *p* = 0.17). This choice-dependent effect on the DPN amplitude differed across both the mediolateral axis (*F*_2,76_ = 36.45, *p* < 0.001, $\eta_{p}^{2}$ = 0.49) and regions (*F*_2,76_ = 10.11, *p* < 0.001, $\eta_{p}^{2}$ = 0.21, main effect of choice x laterality x region interaction: *F*_*1*,76_ = 20.15, *p* < 0.001, $\eta_{p}^{2}$ = 0.35, Figure 4C) so that it was maximal at the midline (left, *F*_*1*,76_ = 59.66, *p* < 0.001, $\eta_{p}^{2}$ = 0.44; midline, *F*_*1*,76_ = 92.41, *p* < 0.001, $\eta_{p}^{2}$ = 0.55; right, *F*_*1*,76_ = 74.43, *p* < 0.001, $\eta_{p}^{2}$ = 0.50) and in frontocentral regions (frontal, *F*_*1*,76_ = 60.30, *p* < 0.001, $\eta_{p}^{2}$ = 0.44; frontocentral, *F*_*1*,76_ = 87.33, *p* < 0.001, $\eta_{p}^{2}$ = 0.53; centroparietal, *F*_*1*,76_ = 74.08, *p* < 0.001, $\eta_{p}^{2}$ = 0.49). Follow-up analyses across all regions except on the left side of the scalp for advantageous choices (*F*_2,76_ = 0.05, *p* = 0.84; all other *Ps* < 0.005).

An exploratory LORETA cortical source estimation suggested that the DPN may originate in the Posterior Cingulate Cortex (PCC, Figure 4D).

#### Feedback processing stage

**Figure 5:**
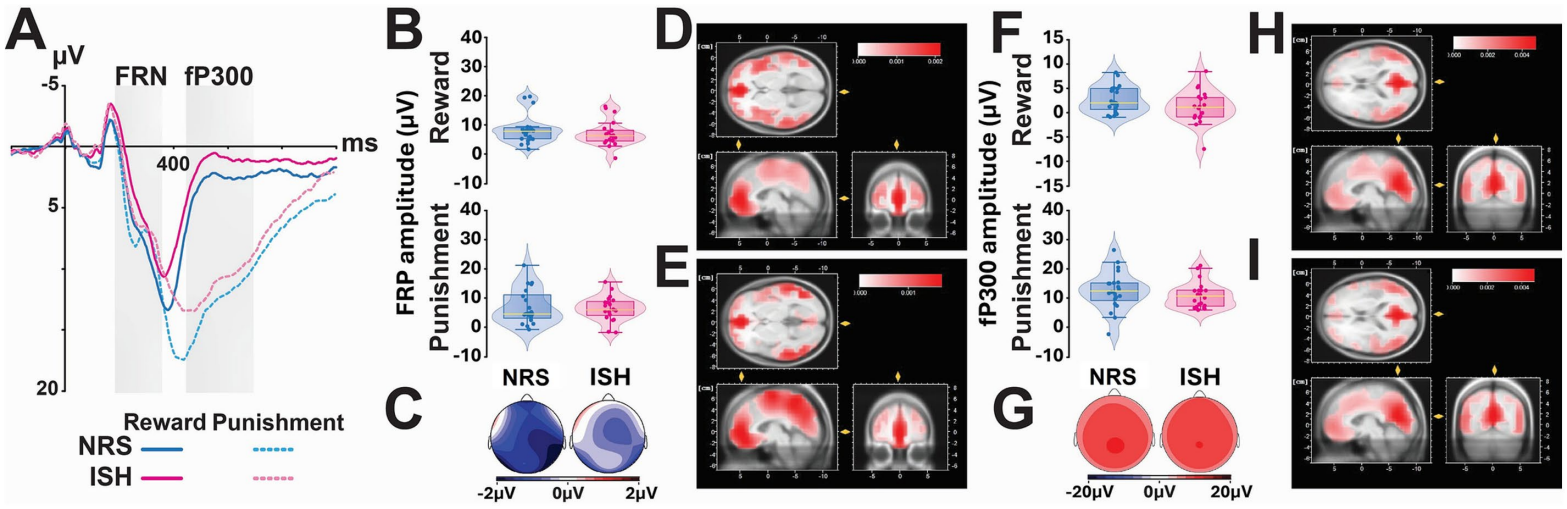
Outcome-related ERP modulations (FRN–ACC, fP300–PCC) differed between negative and positive outcomes during the feedback stage. **(A)** Event-related potentials (ERPs) recorded at Cz for reward (solid line) and punishment (dotted line) are shown for the ISH (blue) and NRS (orange) groups. **(B)** Boxplots depict the mean FRN voltage (μV) at Cz within the 300–360 ms time window accompanied by the corresponding scalp topographies **(C)** and the exploratory LORETA-based cortical source estimations for the outcome effect for ISH **(D)** and NRS **(E)** suggesting that the FRN outcome effect was primarily generated in the anterior cingulate cortex (ACC). **(F)** Boxplots depict the mean fP300 voltage (μV) at Cz within the 450–700 ms time window accompanied by the corresponding scalp topographies **(G)** and the exploratory LORETA-based cortical source estimations for the outcome effect for ISH **(H)** and NRS **(I)** suggesting that the fP300 outcome effect was primarily generated in the posterior cingulate cortex (PCC).

We then investigated the neural correlates of outcome processing. More precisely, we analysed the influence of smoking status and outcome (positive vs. negative) on two ERP components (Figure 5A,C), namely the Feedback Related Negativity (FRN, 300–360 ms; Figures 5B,E) and the fP300 (450–700 ms; Figures 5F–I).

The Feedback Related Negativity (FRN; 300–360 ms) was marginally influenced by the outcome of the choice made by the participants (*F*_1,38_ = 4.25, *p* = 0.08, $\eta_{p}^{2}$ = 0.10), with greater amplitudes elicited after negative than after positive outcomes (4.1 mV ± 4.9 vs. 3.5 mV ± 4.1, respectively). This effect varied across regions (*F*_1,76_ = 14.96, *p* < 0.001, $\eta_{p}^{2}$ = 0.28), being observed in the Centroparietal (*F*_1,76_ = 9.61, *p* < 0.005, $\eta_{p}^{2}$ = 0.11), but not the Frontal (*F*_1, 76_ = 3.16, *p* = 0.08) or the Frontocentral region (*F*_1,76_ = 0.11, *p* = 0.75). This regional distribution of outcome-dependent FRN amplitude was not uniform alongside the mediolateral axis (*F*_4,152_ = 15.04, *p* < 0.001, $\eta_{p}^{2}$ = 0.28) in that it was observed specifically on the right frontocentral region (*F*_1,152_ = 7.60, *p* = 0.009, $\eta_{p}^{2}$ = 0.05) as well as on the right and left side of the centroparietal region (*F*_1,152_ = 18.70, *p* < 0.001, $\eta_{p}^{2}$ = 0.11 and *F*_1,152_ = 15.41, *p* < 0.001, $\eta_{p}^{2}$ = 0.09, respectively; *p* > 0.13 for all other comparisons). These outcome-dependent FRN responses were not influenced by smoking status (*F*_1,38_ = 0.88, *p* = 0.35 and no statistically significant interaction effect involving this factor).

The fP300 (450–700 ms), which follows the FRN, was similarly influenced by the nature of the outcome (*F*_1,38_ = 220, *p* < 0.001, $\eta_{p}^{2}$ = 0.85) with a greater amplitude elicited after negative than after positive outcomes (7.7 μV ± 5.3 vs. 1.0 μV ± 2.7, respectively), but not by smoking status (*F*_1,38_ = 0.11, *p* = 0.74). The outcome-dependent fP300 amplitude modulations were not uniform across regions (*F*_2,76_ = 11.94, *p* < 0.001, $\eta_{p}^{2}$ = 0.24) or the medio-lateral axis (*F*_2,76_ = 36.59, *p* < 0.001, $\eta_{p}^{2}$ = 0.49). Simple main effects analyses revealed that the differences across regions were more significant for negative (F_2,76_ = 11.10, *p* < 0.001, $\eta_{p}^{2}$ = 0.23) than for positive outcomes (F_2,76_ = 3.10, *p* = 0.051, $\eta_{p}^{2}$= 0.08), suggesting that the topographical distribution of the fP300 was more clearly modulated during the processing of negative feedback. Further planned comparisons demonstrated that fP300 amplitudes elicited after negative outcomes were greater on the Frontocentral region than on either the Frontal (*t*_110_ = 5.08, *p* < 0.001, *d* = 0.97) or Centroparietal regions (*t*_110_ = 4.55, *p* < 0.001, *d* = 0.87), between which it did not differ (*t*_110_ = 0.54, *p* = 0.59). Additionally, the fP300 was revealed to be more positive at the centre of the scalp compared to both the left (*t*_76_ = 8.31, *p* < 0.001, *d* = 0.95) and right sides (*t*_76_ = 5.93, *p* < 0.001, *d* = 0.68). This effect was also predicted on the type of outcome (*F*_2,76_ = 82.09, *p* < 0.001, $\eta_{p}^{2}$ = 0.68), being greater for negative (*F*_2,76_ = 55.48, *p* < 0.001, $\eta_{p}^{2}$ = 0.59) than for positive outcomes (F_2,76_ = 9.96, *p* < 0.001, $\eta_{p}^{2}$= 0.21).

An exploratory LORETA cortical source estimation suggested that the Anterior Cingulate Cortex (ACC, Figures 5D,E) may be preferentially activated during the processing of the outcome in the fP300 (300–360 ms) time window whereas the Posterior Cingulate Cortex (PCC, Figures 5H,I) may be preferentially activated during outcome processing in the FRN (450–700 ms) time window.

**Figure 6:**
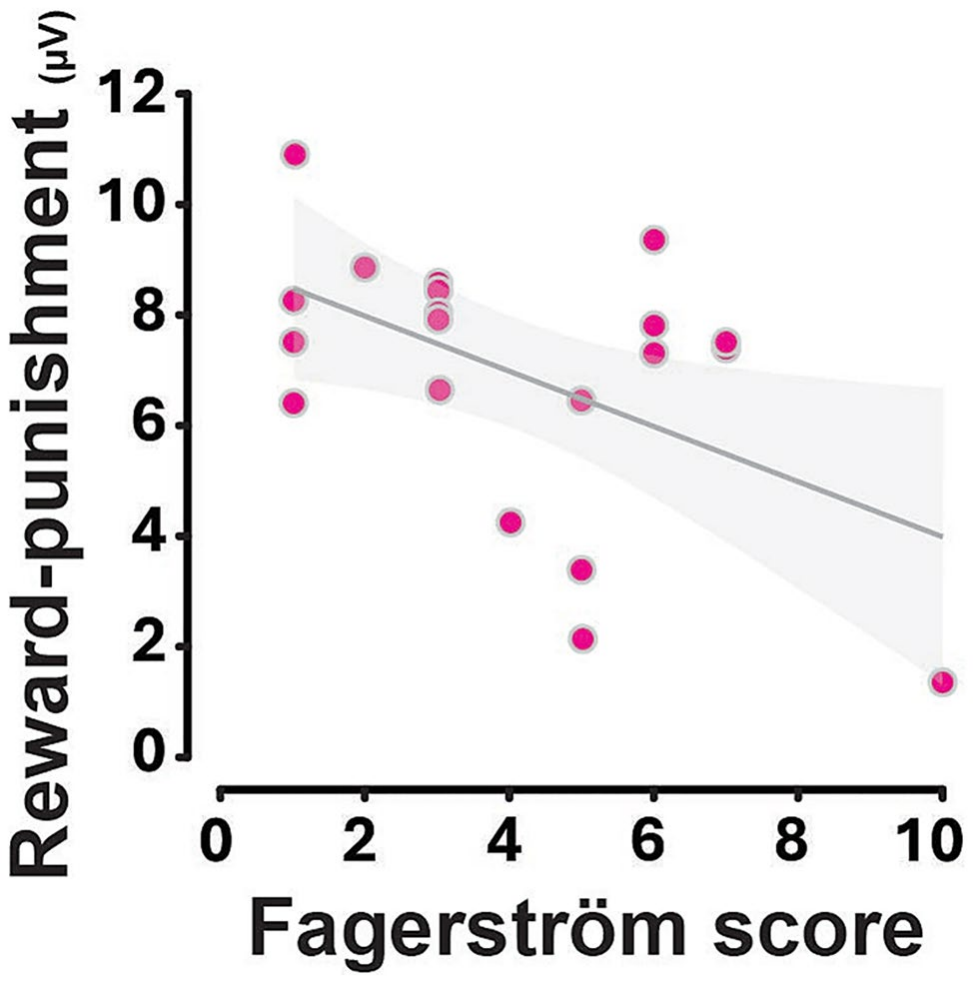
The severity of smoking habits is inversely related to the reward—punishment fP300 amplitude difference. Each data point represents an individual participant. Higher Fagerström scores were associated with a reduced reward—punishment fP300 amplitude difference (*R^2^* = 0.26, *p* = 0.02). The grey line represents the linear regression fit, with the shaded area indicating the 95% confidence interval.

While the smoking status did not influence the fP300 overall, within the ISH, the severity of nicotine dependence shaped fP300-related outcome processing, as revealed by a main outcome x Fagerström scores interaction from an ANCOVA that includes the Fagerström Test scores as a covariate (*F*_1,18_ = 6.15, *p* = 0.02, $\eta_{p}^{2}$ = 0.26). This was further supported by a dimensional analysis which confirmed that in ISH the difference in the fP300 amplitude between positive and negative outcomes was inversely related to the severity of nicotine dependence (*r* = −0.51, *p* = 0.02; Figure 6).

## Discussion

The present ERP study aimed to investigate the neural dynamics at different stages of decision-making in ISH compared to NRS using the Iowa Gambling Task, a paradigm that operationalizes real-life decision-making under uncertainty. At the behavioral level, participants progressively selected more advantageous decks across trials during the IGT session; showing a learning curve that is consistent with previous studies (Bechara et al., 2005). However, only NRS really showed such an adaptive shift towards advantageous choices. Thereby, these results demonstrate that, as anticipated, ISH have a slight decision-making impairment, extending to a population with low to moderate dependence previous findings in individuals with severe substance use disorders (Barry and Petry, 2008; Verdejo-Garcia et al., 2007).

This slight deficit in decision-making in ISH cannot be attributable to their heightened impulsivity, as demonstrated by an ANCOVA. This is consistent with prior work showing weak or non-significant relationships between self-reported impulsivity and behavioural decision-making measures in stimulant users (Hulka et al., 2015). However, since self-reported impulsivity, which requires insight, including interoceptive insight, does not necessarily correlate with behavioural measures of the various subdimensions of impulsivity, in particular in individuals with a substance use disorder (Hulka et al., 2015), further research is necessary to determine whether alterations in specific dimensions of impulsivity, such as motor vs. cognitive impulsivity, as assessed using a continuous performance or delay discounting task, respectively (Robbins and Dalley, 2017), contribute to such an impairment.

Importantly, although the behavioural differences observed between ISH and NRS in this relatively small sample are subtle, analysis of several ERPs revealed more profound neurophysiological differences, underscoring the need to investigate both behavioural and neurophysiological or neurobiological correlates of decision-making in ISH and, more generally, in individuals with a substance use disorder. Considering the substantial heterogeneity in effect sizes and the frequent absence of statistically significant group differences across studies on IGT performance in individuals with a substance use disorder (Gowin et al., 2018), such a combined approach is, we would argue, very useful to elucidate the quantitative and qualitative nature of the altered decision-making function involved in the maintenance of maladaptive drug-related habits.

Thus, to further characterise the neurophysiological correlates of this decision-making impairment, we recorded and analysed the event-related potentials (ERPs) targeting the three processing stages of decision making: outcome evaluation, response selection and outcome processing (or feedback stage). Most ERP studies of SUDs have focused their investigations on the ERP evoked by positive and negative outcomes, consistently demonstrating an attenuated FRN amplitude, indicating reduced sensitivity to negative outcomes (Balconi et al., 2017; Raiha et al., 2020; Zhao et al., 2017; Wei et al., 2021) and an enhanced fP300 following positive outcomes, consistent with heightened motivational salience and striatal reactivity to gains (Euser et al., 2011; Balconi et al., 2017; Zhou et al., 2010; Wei et al., 2018; Luijten et al., 2017). Accordingly in the present study positive and negative outcomes elicited different processing mechanisms at the feedback stage, manifested as outcome-specific modulations of the amplitudes of both the FRN (250–300 ms) and feedback P300 (fP300, 450–700 ms) components. Consistent with prior literature, negative outcomes elicited larger FRN amplitudes than positive outcomes over fronto-central regions. The source estimation (Loreta) identified the ACC as the source of this component, which is consistent with prior findings (Amiez et al., 2005; Bellebaum and Daum, 2008). The FRN is thought to reflect a rapid neural evaluation of outcome valence and prediction errors, being particularly sensitive to unexpected or emotionally salient feedback, which in turn helps guide learning and adaptive behaviour. In that, our results show that ISH and NRS were both able to quickly access outcome valence information. Following the FRN, the fP300 is considered to reflect higher order evaluation processes including attentional allocation and working memory updating. Our exploratory source analysis identified the posterior cingulate cortex (PCC, BA 31) as the source of the activity in the fP300 time window which was stronger for negative than positive outcomes across all participants. This observation is consistent with the established role of that brain structure in valuation, monitoring, and affective evaluation (Leech and Sharp, 2014; Pearson et al., 2011).

Since substance use disorders have been associated with a heightened sensitivity to rewards and a diminished sensitivity to negative outcomes or consequences, we expected that, as compared to NRS, ISH would exhibit reduced FRN and/or fP300 amplitudes evoked by positive compared to negative outcomes. Contrary to our expectations, we observed no main effect of, or interaction involving, smoking status for either the FRN or the fP300. This appears at first glance to be at odds with the outcome of previous ERP studies in SUD populations using the IGT or similar decision-making tasks (Euser et al., 2011; Luijten et al., 2017, Balconi et al., 2017; Raiha et al., 2020; Zhao et al., 2017; Zhou et al., 2010; Wei et al., 2018), which reported attenuated feedback-related potentials in addicted individuals.

However, this apparent discrepancy is likely due to the relatively moderate nicotine dependence and heterogeneity of the ISH group in the present study, as supported by the inverse relationship between fP300 amplitude difference between positive and negative outcomes and nicotine dependence severity, as measured by the Fagerström. Specifically, higher dependence was associated with a reduced differentiation between positive and negative outcomes, a result in line with Morie et al. (2021), which also reported modulations of feedback-related potentials (i.e., FRN latency) both by smoking status and, critically, by the severity of nicotine use in a polydrug user population. Thus, our present results demonstrate in a smoker-only population that greater addiction severity, but not smoking habits per se, blunts the neural differentiation between positive and negative feedback, potentially reflecting an impaired interoceptive insight and the ensuing reduced sensitivity to outcome valence. Whether these alterations reflect a primary impairment in interoceptive insight or compensatory mechanisms remains to be elucidated.

At the neural systems level, exploratory LORETA source estimations suggested a possible differential engagement of the PCC across groups irrespective of outcome valence. This reduced PCC engagement in smokers may reflect diminished integration of outcome value into self-referential or motivational processes, a deficit has been hypothesised to underlie the blunted capacity to learn from negative consequences in addiction (Broyd et al., 2009; Garavan et al., 2008). Prior fMRI work has also implicated PCC hypoactivity in impaired cognitive control and craving regulation in SUD populations (Goldstein and Volkow, 2011). Together, these results suggest that altered PCC function could be a neural correlate of sub-optimal outcome-based learning and motivational adjustment in ISH. However, given the absence of significant ERP amplitude differences between groups, these source-level observations should be interpreted cautiously and considered as exploratory spatial characterizations that may help guide future hypothesis-driven investigations.

Another objective of the present study was to investigate ERPs elicited prior to response production, which relate to two largely unexplored stages: choice evaluation and response selection (Latibeaudiere et al., 2025). At the choice evaluation stage, locked on decks presentation, the amplitude of the P300 (390–450 ms) and the N500 (480–520 ms) were shown to be sensitive to the selection of risky or disadvantageous options, in agreement with the literature. Specifically, we observed that disadvantageous choices elicited larger P300 and N500 responses, particularly over right centro-parietal and fronto-central sites, suggesting the engagement of attention- (Yang et al., 2007; Yang et al., 2010), and emotion- or motivation-relevant neural networks (Guo et al., 2019) engaged in evaluating advantageous vs. disadvantageous options. Importantly, ISH exhibited greater P300 amplitudes than NRS in frontal parts of the scalp irrespective of the subsequent choice. Exploratory LORETA cortical source estimation suggested that this differential activity originates in the orbitofrontal cortex.

Interestingly, the amplitude of the N500 was also modulated by smoking status, but this effect was restricted to advantageous choices only, thereby suggesting an asymmetric two-stage evaluation process in ISH as compared to NRS. Exploratory LORETA cortical source estimation suggested that this asymmetry may reflect a differential involvement of the PCC and the frontal midline gyrus in NRS and ISH, respectively.

Altogether these observations support the hypothesis that ISH rely on a different strategy to evaluate options, characterised by an excessive engagement of attentional mechanisms rather than reliance on somatosensory information. Specifically, ISH over-engage attentional and motivational mechanisms for risky options while under-optimally allocating conflict monitoring resources for advantageous choices. This imbalance could reflect a deficit in integrating somatic markers into decision-making in ISH, consistent with the somatic marker theory of addiction (Verdejo-Garcia and Bechara, 2009).

During the response selection stage, locked on the response made by the participants, Decision Preceding Negativity (DPN, −800–0 ms) was shown to be more pronounced prior to disadvantageous, or risky, choices, consistent with previous findings indicating that this component reflects anticipatory mechanisms engaged before committing to a risky decision (Latibeaudiere et al., 2025; Bianchin and Angrilli, 2011; Dong et al., 2016; Cui et al., 2013). This enhanced negativity likely indexes the recruitment of cognitive control and preparatory processes, as participants evaluate potential risks and attempt to regulate forthcoming actions.

No effect of smoking status was observed on DPN amplitude, in line with previous evidence that the DPN reflects general risk anticipation rather than individual variability in task performance (Giustiniani et al., 2015). Importantly, these results together suggest that ISH, while showing alterations in early choice evaluation (P300/N500) and feedback processing (fP300) stages, may retain relatively preserved anticipatory mechanisms when preparing to execute a decision.

While the samples used in the present study may not be large enough for subtle differences between groups to systematically reach statistical significance according to current norms (see Figure 3F), overall, the effect sizes of the differences observed are large to very large, and the results, which show a very strong internal consistency, align well with the literature.

## Conclusion

Altogether, the results of this study highlight a dual imbalance in smokers’ decision-making with an over-engagement of attention and motivational processes towards risky or disadvantageous options on one side, and a reduced sensitivity to outcome valence, especially when nicotine dependence is more severe, on the other side. Difficulties in discriminating between outcomes have been documented across several SUDs, including cocaine (Goldstein et al., 2007; Goldstein et al., 2007) and nicotine (Potts et al., 2014), and have been hypothesised to contribute both to maladaptive decision-making and a reduced capacity to sustain motivation towards long-term goals (Goldstein and Volkow, 2011). This pattern observed here in ISH is potentially associated with a differential engagement of the PCC and the frontal midline gyrus may represent a neural mechanism underlying the persistence of smoking behaviour despite awareness of long-term negative consequences in ISH.

## Data Availability

The raw data supporting the conclusions of this article will be made available by the authors, without undue reservation.
